# A novel missense variant in *ESRRB* gene causing autosomal recessive non-syndromic hearing loss: in silico analysis of a case

**DOI:** 10.1186/s12920-022-01165-4

**Published:** 2022-02-01

**Authors:** Tohid Ghasemnejad, Mahmoud Shekari Khaniani, Jafar Nouri Nojadeh, Sima Mansoori Derakhshan

**Affiliations:** 1 Neurosciences Research Center, Tabriz University of Medical Sciences, Tabriz, Iran; 2 Department of Medical Genetics, Faculty of Medicine, Tabriz University of Medical Sciences, Tabriz, Iran; 3 Molecular Medicine Research Center, Biomedicine Institute, Tabriz University of Medical Sciences, Tabriz, Iran

**Keywords:** Hearing loss, Consanguineous marriage, NGS, *ESRRB*, ARNSHL, Iran

## Abstract

**Background:**

Hereditary hearing loss (HHL) is a common heterogeneous disorder affecting all ages, ethnicities, and genders. The most common form of HHL is autosomal recessive non-syndromic hearing loss (ARNSHL), in which there is no genotype–phenotype correlation in the majority of cases. This study aimed to identify the genetic causes of hearing loss (HL) in a family with Iranian Azeri Turkish ethnicity negative for gap junction beta-2 (*GJB2*), gap junction beta-6 (*GJB6*), and mitochondrially encoded 12S rRNA (*MT-RNR1*) deleterious mutations.

**Methods:**

Targeted genome sequencing method was applied to detect genetic causes of HL in the family. Sanger sequencing was employed to verify the segregation of the variant. Finally, we used bioinformatics tools and American College of Medical Genetics and Genomics/Association for Molecular Pathology (ACMG/AMP) guidelines to determine whether the detected variant might affect the corresponding protein or not.

**Results:**

A novel homozygous missense mutation, c.499G>A (p.G167R), was identified in exon 5 of the *ESRRB* (estrogen-related receptor beta) gene. Healthy and affected family members confirmed the co-segregation of the variant with ARNSHL. Eventually, the variant's pathogenicity was confirmed by the in silico analysis and the ACMG/AMP guidelines.

**Conclusion:**

The study suggests that the detected variant, c.499G>A, plays a crucial role in the development of ARNSHL, emphasizing the clinical significance of the *ESRRB* gene in ARNSHL patients. Additionally, it would be helpful for genetic counseling and clinical management of ARNSHL patients and providing preventive opportunities.

**Supplementary Information:**

The online version contains supplementary material available at 10.1186/s12920-022-01165-4.

## Background

Hereditary hearing loss (HHL) is one of the hereditary disorders and a public health concern, affecting approximately one in 650–1000 newborns in developed countries [1]. Although environmental factors can act as a trigger, the role of genetic factors is significant in the etiology of HL [2]. Current knowledge shows that approximately 70% of all HHL cases are in the form of non-syndromic, in which hearing loss (HL) is the only clinical symptom, while syndromic forms account for 30% of the cases in which HL is part of other syndromes [3]. Besides, HHL can be transmitted as autosomal dominant (20%), autosomal recessive (70–80%), X-linked (2–5%), and mitochondrial (1%) inheritance [4]. Based on genomic variation databases, including human gene mutation database (HGMD), ClinVar, and single nucleotide polymorphism database (dbSNP), more than 2000 variants have been determined in non-syndromic hearing loss (NSHL). Homozygous or compound heterozygous variants in the gap junction beta-2 (*GJB2*) gene and large deletions in the gap junction beta-6 (*GJB6*) gene are the common causes of HL in Iran and many other countries [5–7]. Furthermore, according to previous studies in Iran, mutations in the solute carrier family 26 member 4 (*SLC26A4)* gene [8] is another common cause of NSHL followed by mutations in Myosin XVA (*MYO15A*) [9] Myosin VIIA (*MYO7A*) [7], Cadherin related 23 (*CDH23*) [10], Protocadherin related 15 (*PCDH15*) [10], alpha-Tectorin (*TECTA*) [11], Pejvakin (*PJVK*) [12], transmembrane channel-like protein 1 (*TMC1*) [13], Leucine-rich transmembrane and O-methyltransferase domain-containing (*LRTOMT*) [14], immunoglobulin-like domain-containing receptor 1 (*ILDR1*) [15], MARVEL domain containing 2 (*MARVELD2*) [7], Otoferlin (*OTOF*) [7], Radixin (*RDX*) [7], Lipoxygenase homology PLAT domains 1 (*LOXHD1*) [16], and Collagen type XI alpha 2 chain (*COL11A2*) genes [17]. According to Bazazzadegan et al. study in Iran, the prevalence of *GJB2* mutations varies based on geographical location and ethnicity; for example, in Azerbaijan provinces (northwest of Iran where our study was done), *GJB2* mutations account for 22% of HL cases whereas, in Sistan and Baluchestan province (southeast of Iran with Baluch ethnicity), this amount is only 8% [7].

The limited phenotypic variability and high heterogeneity of NSHL make it particularly difficult to diagnose by routine detection methods [5]. Recent advances in diagnostic methods such as next-generation sequencing (NGS) have opened a new window for heterogenic disorders; however, the production of thousands of variants per individual is the biggest challenge of NGS technology. Especially when directing clinical care, there is a need for correct interpretation and ethnic-specific filtering of test results to achieve precise outcomes [17–20]. In this light, this study aimed to employ the targeted sequencing of 127 known HL-causing genes panel in the proband of a family of Iranian Azeri Turkish ethnicity with consanguine marriage negative for *GJB2*, *GJB6,* and mitochondrially encoded 12S rRNA (*MT-RNR1*) mutations.

## Methods

### Participants

**Fig. 1:**
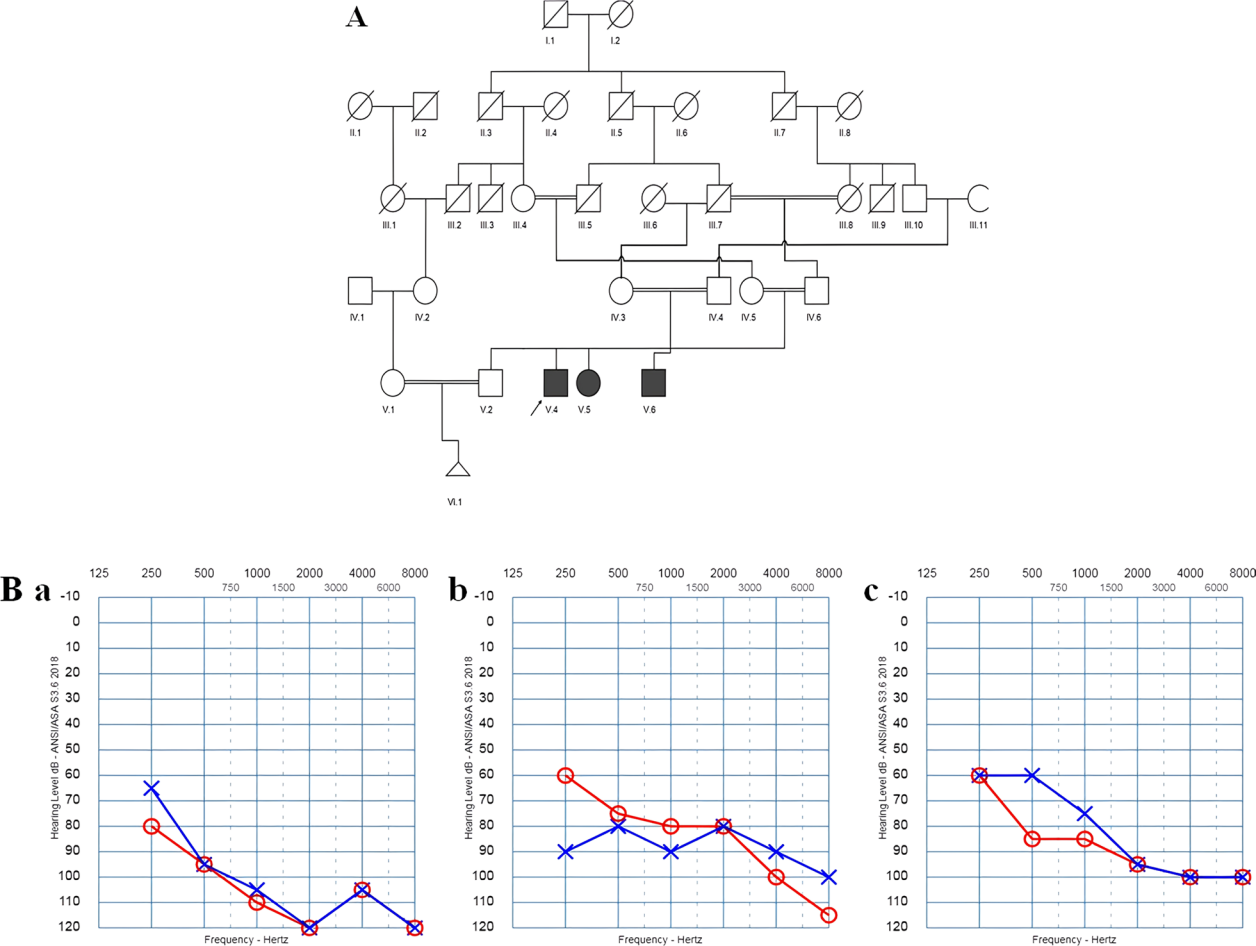
(**A**) The pedigree shows a consanguineous marriage between parents, which results in the inheritance of autosomal recessive non-syndromic hearing loss in their children. (**B**) The audiograms show bilateral severe to profound hearing loss in (a) V4, (b) V5, and (c) V6

An affected Iranian individual of Azeri Turkish ethnicity, whose parents had a consanguineous marriage (Fig. 1A), was recruited from Ebnsina Medical Genetic Laboratory of Tabriz. The proband originated from Tabriz, a city in the northwest of Iran with majorly Azeri Turkish ethnicity [21]. Physical examination, audiological evaluation, clinical questionnaires on age, exposure to environmental factors, and history of other diseases were administered when the patient was being tested for *GJB2*, *GJB6*, and *MT-RNR1* (A1555G) mutations. To evaluate the detected variant in the control population, 200 blood samples were collected in the same region (Tabriz). The majority (64%) of this population were male, with a mean age of 36 years and no history of HLL. Full informed consent was obtained from the participants in the study. The Ethics Committee of Tabriz University of Medical Sciences approved the study and methodology for investigating humans with ethical code No. 94/2-7/13.

### Targeted genomic capturing and next-generation sequencing

Genomic DNA was extracted from 3 ml peripheral blood of the patient using a DNA Extraction Mini Kit (FavorPrep, Taiwan, Cat. No.: FATGK001). Then, the quality and quantity of the DNA were determined with a spectrophotometer and a 1.2% agarose gel. We sent the DNA sample to the Beijing Genomics Institute (BGI-Clinical Laboratories, Shenzhen, China) to perform the targeted sequencing. The sequencing test was conducted using a custom-designed Nimblegen chip capturing the 127 targeted genes, followed by next-generation sequencing. The test platform examined > 95% of the target gene with a sensitivity of > 99%. Point mutation, micro-insertion, deletion, and duplication (< 20 bp) could be simultaneously detected. Illumina Base Calling software (bcl2fastq) was used to analyze the raw data. Human genome assembly hg19 (GRCh37) was used as the reference sequence. A single nucleotide variant detection toolkit (GATK v3.3.0) was utilized to detect single nucleotide variants (SNV/INDEL), and then the variants were annotated using ANNOVAR software. In addition, all variants were then filtered according to the mutations' type, frequency, and pathogenicity.

### Variant confirmation by sanger sequencing

Primers surrounding the damaging variant were designed using Primer 3 software (forward: GCCACCAGGCTAAATCCTCTG and reverse: TTTCCAAGGTCACCAGGCAGT) to evaluate the variant associated with the phenotype. After amplification by an Eppendorf thermocycler and using a SinaClon PCR Master Mix kit (Cat. No.: MM2011), Sanger sequencing was performed utilizing a Big Dye kit (Cat. No.: 4337455) and an Applied Biosystems instrument (3130xl Genetic Analyzer). Finally, Chromas software version 2.6.4 was used to observe the obtained sequences. In order to assess the detected variant in the control population, we extracted genomic DNA from the 200 blood samples using salting-out procedure. We then performed PCR amplification and Sanger sequencing using primers surrounding the candidate gene to obtain the sequences.

### Functional prediction and molecular modeling

For the pathogenicity prediction of the identified variant, multiple bioinformatics software and web servers were used, including Polyphen (http://genetics.bwh.harvard.edu/pph2/), SIFT (https://sift.bii.a-star.edu.sg/), FATHMM (http://fathmm.biocompute.org.uk/), MetaSVM (https://omictools.com/metasvmtool) LRT (http://www.genetics.wustl.edu/jflab/lrt_query.html), MutationAssessor (http://mutationassessor.org/r3/), MutationTaster (http://www.mutationtaster.org/), LOFtool (https://github.com/konradjk/loftee) and GERP^++^ (https://github.com/tvkent/GERPplusplus). For conservation analysis, we used UniProtKB/UniRef100 web server (http://www.uniprot.org/uniref/UniRef100) for aligning protein sequences to evaluate the conservation and evolutionary relationships among vertebrate species.

The effect of the mutation on the molecular level was analyzed by modeling software Yet Another Scientific Artificial Reality Application (YASARA) (http://www.yasara.org/). The ESRRB protein model used for this analysis was according to the PDB file with an ID of 1lo1.

The impact of amino acid substitution on the ESRRB protein structure stability was analyzed using MAESTROweb (https://biwww.che.sbg.ac.at/maestro/web/maestro/workflow) and SDM (Site Directed Mutator) (http://www.structure.bioc.cam.ac.uk/sdm2) web servers. Additionally, we used STRING (Search Tool for the Retrieval of Interacting Genes/Proteins) database (https://string-db.org/) to predict protein–protein interactions between ESRRB and other proteins.

Finally, we used minor allele frequency (MAF) to discriminate common polymorphism from rare variants in the population databases, and the MAF < 1% is considered a rare variant [5]. The employed population databases were 1000 Genomes Project (http://www.1000genomes.org/), Exome Sequencing Project (http://evs.gs.washington.edu/EVS/), and Exome Aggregation Consortium (http://exac.broadinstitute.org).

## Results

### Clinical evaluations

In this family, both parents had normal hearing levels. A three-year-old boy was the proband (IV.3). In addition to him, both affected members of the family (V.5 and V.6) displayed bilateral severe to profound HL (Fig. 1a–c). As stated by parents, HL was congenital, and no other abnormality indicated syndromic HL. As much as remembered by the mother, there was no exposure to environmental factors, and no special disease would have made use of a particular medicine or antibiotic such as aminoglycosides.

### Functional prediction and molecular modeling

**Fig. 2:**
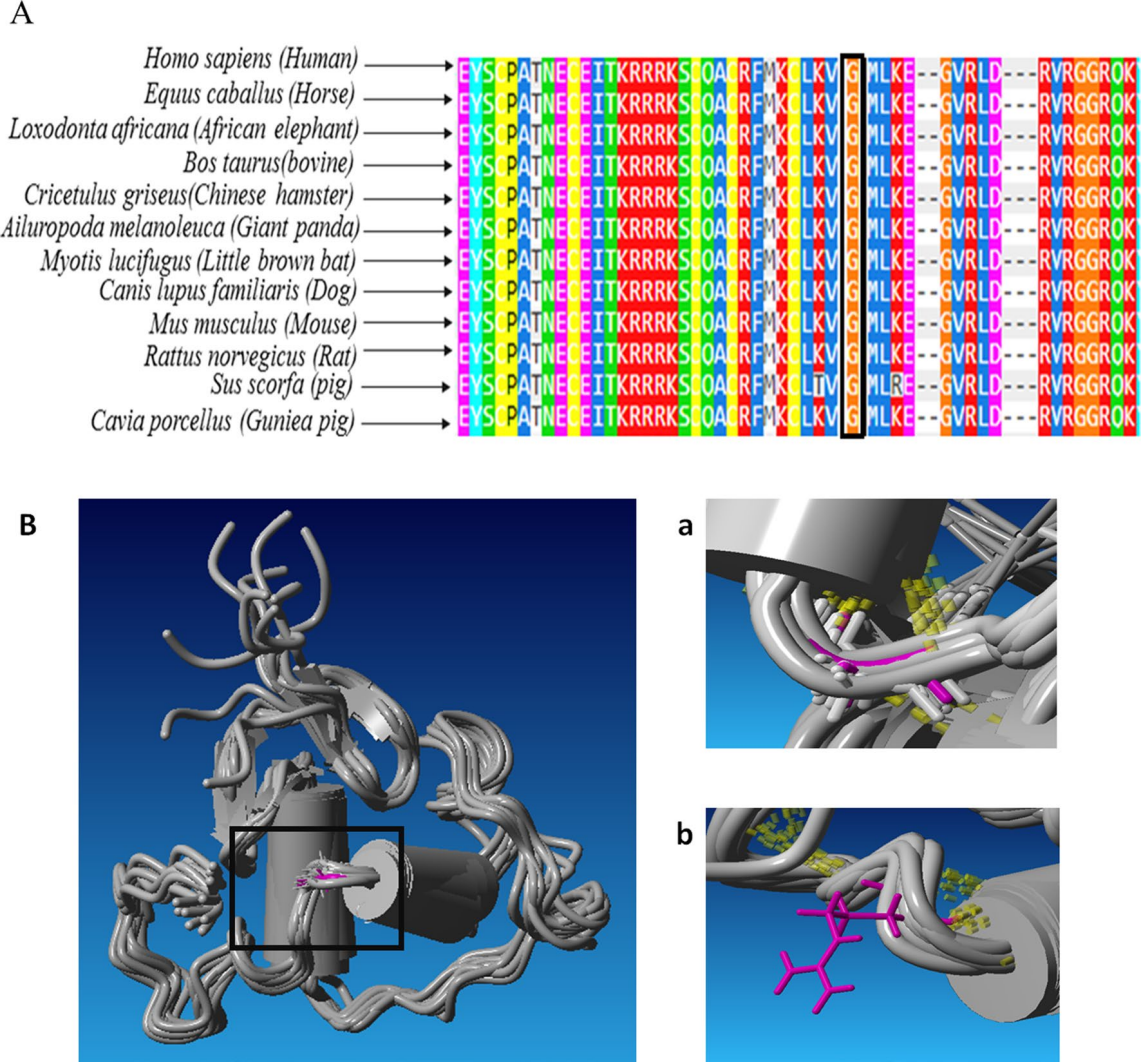
(**A**) Visualization of multiple sequence alignment of the ESRRB protein in mammals including the missense mutation site. The location of the altered amino acid has been indicated in the black box. (**B**) Molecular modelling of the ESRRB protein. The wild-type residue (Gly167) of the ESRRB protein has been depicted with violet. **(a)** Molecular modeling predicted hydrogen interaction between Gly167 and Ala163 residues. Hydrogen bonds have been shown with the yellow dotted line. **(b)** the large side chain of Arg in the 167th position possibly increases inappropriate interaction between residues

**Fig. 3:**
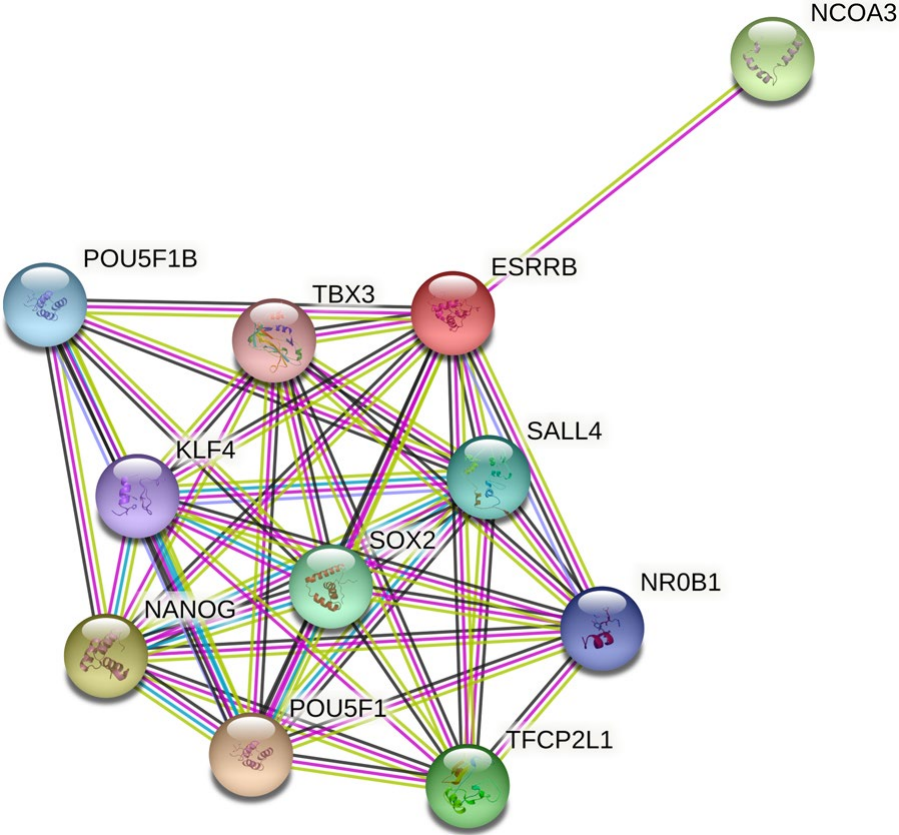
Schematic representation of protein–protein interaction network of ESRRB using STRING database. Predicted functional partners are as follows: NCOA3 (Nuclear receptor coactivator), TBX3 (T-box transcription factor), POU5F1 (Putative POU domain, class 5, transcription factor 1B), SALL4 (Sal-like protein 4), NR0B1 (Nuclear receptor subfamily 0 group B member 1), TFCP2L1 (Transcription factor CP2-like protein 1), POU5F1 (POU domain, class 5, transcription factor 1), NANOG (Homeobox protein NANOG), KLF4 (Krueppel-like factor 4) and SOX2 (SRY-Box Transcription Factor 2)

The targeted genomic capturing in this family indicated a novel homozygous variant c.499G>A (p. Gly167Arg) in the *ESRRB* gene (estrogen-related receptor beta-NM_004452.1), submitted by our group with Ref SNP ID: rs155534214. The variant was co-segregated with the ARNSHL phenotype (Additional File 1) and was absent in 200 control individuals (data not shown). Based on the results of pathogenicity prediction, the c.499G>A variant was predicted as a damaging variant by all databases (Table 1), and more importantly, it had no frequency in population databases (Table 1). The conservation evaluation of the 167th amino acid of the ESRRB protein indicated that glycine was a conserved amino acid (Fig. 2A), where substituted arginine caused a large side chain in the protein and could destabilize it (Fig. 2B). Interactome analysis using the STRING database showed that the ESRRB protein interacted with the following proteins (Fig. 3): NCOA3 (Nuclear receptor coactivator), TBX3 (T-box transcription factor), POU5F1 (Putative POU domain, class 5, transcription factor 1B), SALL4 (Sal-like protein 4), NR0B1 (Nuclear receptor subfamily 0 group B member 1), TFCP2L1 (Transcription factor CP2-like protein 1), POU5F1 (POU domain, class 5, transcription factor 1), NANOG (Homeobox protein NANOG), KLF4 (Krueppel-like factor 4), and SOX2 (SRY-Box Transcription Factor 2).

**Table 1:** Characteristics and the pathogenicity prediction of the identified variant

Features of the reported variant	
Gene symbol	*ESRRB*
Locus	DFNB35
Nucleotide acid change	c.499G>A
Ref SNP Id	rs1555342141
Protein change	p.G167R
Domain	DBD
Co-segregation	Yes
Frequency in the control population of this study	0.000
ACMG/AMP criteria	PM5, PM1, PM2, PP1, PP3

	Variant pathogenicity prediction	
	Web servers	Values
Pathogenicity	PolyPhen2	Probably damaging (0.997)
	SIFT	Deleterious (0.0)
	FATHMM	Damaging (− 6.21)
	MutationAssessor	High (3.65)
	MutationTaster	Disease-causing (1.000)
	LOFtool	Probably damaging (0.193)
	MetaSVM	Deleterious (0.911)
	LRT	Deleterious (0.62918)
	GERP++	Damaging (5.12)
MAF	1000 Genomes	0.000
	ESP	0.000
	ExAC	0.000
Conservation	UniProtKB/UniRef100	Yes
Protein stability	SDM	Destabilizing
	MAESTROweb	Destabilizing

The Polymorphism phenotyping v2 (PolyPhen-2) scores range from 0.0 (tolerated) to 1.0 (deleterious). The Sorting Intolerant from Tolerant (SIFT) values range from 0 to 1. The variant is predicted damaging if the score is ≤ 0.05, and tolerated if the score is > 0.05. The Functional Analysis through Hidden Markov Models (FATHMM) scores range from − 16.13 to 10.64. The smaller the score the more likely the variant has a damaging effect. If a FATHMM score is ≤  − 1.5 the corresponding variant is predicted as "D(AMAGING)"; on the contrary, it is predicted as "T(OLERATED)". MutationAssessor scores range from 5.135 to 6.49. MutationAssessor's functional impact of a variant: predicted functional, i.e. high ("H") or medium ("M"), or predicted non-functional, i.e. low ("L") or neutral ("N"). MutationTaster score ranges from 0 to 1 and a larger score means more likely to be deleterious. MutationTaster predictions are "A" ("disease_causing_automatic"), "D" ("disease_causing"), "N" ("polymorphism") or "P" ("polymorphism_automatic"). The Loss-of-function (LOF) tool scores < 0.7 are considered benign, scores < 0.2 are considered probably damaging and a score of 0.2 to 0.7 are possibly damaging. The Meta Support Vector Machine (MetaSVM) score ranges from − 2 to 3 and the larger scores indicate the variant is more likely to be damaging. The likelihood ratio test (LRT) ranges from 0 to 1 and the LRT predictions are D(eleterious), N(eutral), or U(nknown). Genomic Evolutionary Rate Profiling (tmGERP)++ scores range from − 12.3 to 6.71, where a larger score indicates deleterious variation. ACMG/AMP, American College of Medical Genetics and Genomics/Association for Molecular Pathology; MAF, Minor Allele Frequency; ESP, Exome Sequencing Project; ExAC, Exome Aggregation Consortium; SDM, Site Directed Mutator; SNP, Single Nucleotide Polymorphism

## Discussion

The *GJB2* mutations are the most common cause of deafness worldwide, including in Iran [7, 22]. This gene is responsible for about 16–18% of ARNSHL cases in Iran; however, there is a significant gap in recognition of other responsible genes [23]. Considering the heterogeneity of ARNSHL and the high prevalence of HL combined with the high frequency of consanguineous marriages in Iran, it is crucial to identify any involvement of other responsible genes associated with this phenotype [23, 24]. In this study, we used the targeted genomic capturing method and found a variant in the *ESRRB* gene causing ARNSHL.

The *ESRRB* gene, consisting of 11 exons, codes the ERRRB protein. It is well established that the defective ESRRB protein is strongly associated with HL in the mice model [25]. The ESRRB protein belongs to a family of orphan nuclear receptors with broad expression profiles, playing a generic role in regulating energy metabolism in mammals. In addition, this protein enhances self-renewal and reprogramming ability in cells, and a lack of ESRRB function has been shown to impair germ cell development [26]. This protein consists of two critical domains, including ligand-binding domain (LBD) and DNA-binding domain (DBD), which are recruited as the transcriptional regulators of estrogen target genes [25]. The LBD is located at the C-terminal of the ESRRB protein and is organized in a three-layer sandwich structure, containing 12 α-helices (H1-H12). This complex structure contributes to the structural stability and binding ability of the ESRRB protein and plays an essential role in the activation of transcription. The DBD is located in the N-terminal portion of the ESRRB protein, which contains a zinc finger domain and is responsible for DNA binding [27].

**Fig. 4:**
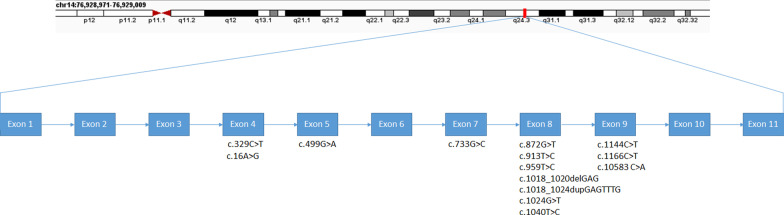
Schematic illustration of the *ESRRB* gene mRNA transcript and the reported mutations so far

**Fig. 5:**
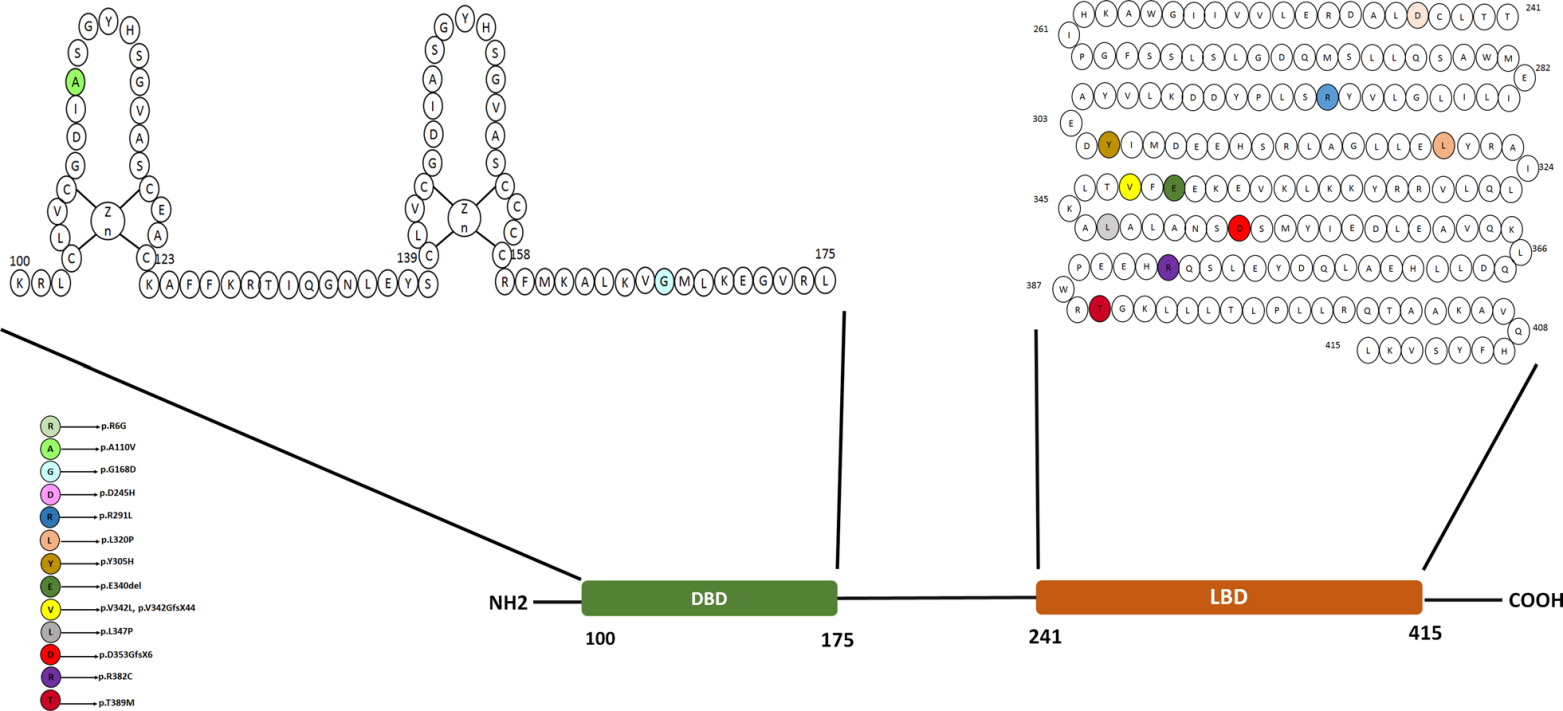
Structure, domains, and the reported mutations in the ESRRB protein

The *ESRRB* gene is the only known gene that acts as a transcription factor and is associated with ARNSHL, while other transcription factors are related to autosomal dominant non-syndromic hearing loss (ADNSHL) [28]. This gene is responsible for the recessive DFNB35 deafness forms. The DFNB35 locus was identified in 2003 when NSHL was being studied in a large Pakistani family [29]. There are currently more than 13 variants have been found in DFNB35, nearly half of which relates to Pakistani ethnicity (p.A110V, p.L320P, p.E340del, p.V342L, p.L347P, p.D245H) [25, 30, 31] and the rest belongs to the Czech Republic (p.R291L) [32], Tunisia (p.Y305H) [28], Turkey (p.V342GfsX44) [25], China (p.R382C, p.R6G) [33, 34], the UAE (p.D353GfsX6) [35], and our discovered variant (p.G167R) in the Iranian population. Except for c.1156C>T [25] and c.1237G>A [32], which are reported as polymorphism, other variants are considered functional mutations that interfere with the function of the ESRRB protein (Table 2). As shown in Fig. 4, most of the variants are accumulated in exon 8 of the *ESRRB* gene at the mRNA level, causing them to appear in the ligand-binding domain of the protein, as shown in Fig. 5. In this report, the sequence analysis of the *ESRRB* gene showed a missense variant c.499G>A, which occurred in exon 5 of the *ESRRB* gene and caused the substitution of glycine by arginine at position 167 in the DBD of the ESRRB-coded protein. This is the first mutation of the *ESRRB* gene identified in an Iranian ethnic group and can be considered a rare cause of ARNSHL in this population. The American College of Medical Genetics and Genomics/Association for Molecular Pathology (ACMG/AMP) has published guidelines for the clinical interpretation of variants providing 28 criteria for classifying a variant as pathogenic/likely pathogenic (P/LP) or benign/likely benign (B/LB) [36]. According to the ACMG/AMP guidelines, our discovered variant met the pathogenicity criteria and was considered “likely pathogenic” because it is a novel missense variant (PM5) within a functional domain (PM1). Additionally, this variant was absent in the controls (PM2), co-segregated in the pedigree (PP1), and computational analysis supports the damaging effects of the variant (PP3).

**Table 2:** An overview of the *ESRRB* mutations reported to date for non-syndromic autosomal recessive hearing loss

Nt change	Aa change	Exon position	Variation type	Zygosity	Domain	Severity	Country	Ref
c.16A>G	p.R6G	4	Missense	Homo/Hetero	N-terminal	ND	China	[33]
c.329C>T	p.A110V	4	Missense	Homo	DBD	S to P	Pakistan	[25]
c.499G>A	p.G167R	5	Missense	Homo	DBD	S to P	Iran	This study
c.733G>C	p.D245H	7	Missense	Homo	LBD	M to S	Pakistan	[31]
c.872G>T	p.R291L	8	Missense	Homo	LBD	P	The Cz.Rep	[32]
c.913T>C	p.Y305H	8	Missense	Homo	LBD	P	Tunisia	[28]
c.959T>C	p.L320P	8	Missense	Homo	LBD	S to P	Pakistan	[25]
c.1018_1020delGAG	p.E340del	8	Frame-shift	Homo	LBD	S to P	Pakistan	[30]
c.1018_1024dupGAGTTTG	p.V342GfsX44	8	Frame-shift	Homo	LBD	S to P	Turkey	[25]
c.1024G>T	p.V342L	8	Missense	Homo	LBD	S to P	Pakistan	[25]
c.1040T>C	p.L347P	8	Missense	Homo	LBD	S to P	Pakistan	[25]
c.1144C>T	p.R382C	9	Missense	ND	LBD	ND	China	[34]
c.1166C>T	p.T389M	9	Missense	Hetero	LBD	ND	Turkey	[25]
c.1058-3C>A	p.D353GfsX6	Donor site of exon 9	Splice site	ND	LBD	ND	The UAE	[35]

*Nt* nucleotide, *AA* amino acid, *Homo* homozygous, *Hetero* heterozygous, *S* severe, *P* profound, *M* moderate, *ND* not determined, *DBD* DNA-binding domain, *LBD* ligand-binding domain, *UAE* United Arab Emirates, *CZ.Rep* Czech Republic

Multiple sequence alignment of the human ESRRB protein across other species showed that the 167th amino acid was located in the highly conserved position at DBD, and it seems that an amino acid change in this position interfered with the function of the ESRRB protein. Molecular modeling prediction revealed that substituting aspartic acid with a glycine residue would lead to a large side chain, possibly resulting in inappropriate interactions between residues. This abnormality is likely to destroy the 3D structure of the DBD and subsequently impair the ESRRB protein's ability to bind to DNA. Due to the comparable clinical phenotype of missense and frameshift mutations, it can be recommended that the molecular mechanism underlying HL is similar and possibly is caused by a loss of function. This idea is supported by Collin et al.’s study of conditional ESRRB^−/−^ mice, in which defective hearing was accompanied by defective ion homeostasis and endolymph production [25]. In another study, Chen et al. found that conditional ESRRB^−/−^ mice were significantly deficient in vestibular function, and their endolymph production decreased. In addition, they showed a reduction in the expression of ion channels in the inner ear, highlighting the importance of ESRRB in inner ear homeostasis [33]. Furthermore, the ESRRB is also essential for functioning and developing inner ear cells through its interaction with estrogen, glucocorticoids, and thyroid hormones. These hormones are essential for inner ear cells so that ESRRB defects can influence cellular processes within the inner ear [25]. Even though ESRRB plays an important role in developing inner ear cells, the exact molecular mechanisms by which it affects other target proteins remains unclear. Nevertheless, Colin et al. observed specific patterns in the distribution of ESRRB transcripts in the developing mouse inner ear during embryonic development [25]. Our interactome analysis (Fig. 3) confirmed their results, which revealed ESRRB interacts with most embryogenesis proteins (such as Nanog, SOX2, KLF4, etc.), and their activities might be influenced by each other. Unraveling these regulatory mechanisms is critical to a thorough understanding of HL caused by ESRRB. Altogether, our study confirmed the pathogenicity of the identified* ESRRB* mutation by computational analysis. However, as a significant limitation, the functional consequences of the mutations were not assessed in this study, which would reveal the specific mechanism by which the c.499G>A mutation affects the ESRRB structure.

## Conclusion

Our study evaluated the genetic cause of bilateral severe to profound HL in an Iranian family with Azeri Turkish ethnicity. Using a targeted gene panel, we identified a novel missense variant in the *ESRRB* gene, which has not already been reported in any literature or databases. Our study confirmed the diagnosis of c.499G>A in the *ESRRB* gene at the molecular level and verified its pathogenicity by in silico analysis and the ACMG/AMP guidelines. It is worth mentioning that this report emphasizes the significance of consanguinity marriage, a phenomenon common in Iran that causes homozygous mutations in these types of disorders. Moreover, reporting novel variations can enhance the clinical management process and genetic counseling, thus offering opportunities to prevent the diseases.

## Supplementary Information

**Additional file 1:** Forward and reverse reads obtained from sequencing of the (**a**) proband (V.4) (**b**) father (IV.6) (**c**) mother (IV.5) and siblings which include (**d**) affected sister (V.5) and (**e**) unaffected brother (V.2).

## Data Availability

The datasets generated and/or analyzed during the current study have been uploaded in the NCBI dbVar repository (https://www.ncbi.nlm.nih.gov/projects/SNP/snp_ss.cgi?subsnp_id=ss2137543931). The accession number for the variant submitted in ClinVar is SCV001739271 and available at https://www.ncbi.nlm.nih.gov/clinvar/submitters/508148/.

## Abbreviations

ER: Estrogen receptors
HGMD: Human Gene Mutation Database
NGS: Next-generation sequencing
HHL: Hereditary hearing loss
HL: Hearing loss
DBD: DNA-binding domain
ARNSHL: Autosomal recessive non-syndromic hearing loss
MT-RNR1: Mitochondrially encoded 12S rRNA
HHL: Hereditary hearing loss
YASARA: Yet another scientific artificial reality application
SDM: Site-directed mutator
MAF: Minor allele frequency
LBD: Ligand-binding domain
GJB2: Gap junction beta-2 protein
GJB6: Gap junction beta 6 protein
SLC26A4: Solute carrier family 26 member 4
MYO15A: Myosin XVA
MYO7A: Myosin VIIA
CDH23: Cadherin related 23
PCDH15: Protocadherin related 15
TECTA: Alpha-Tectorin PJVKPejvakin
TMC1: Transmembrane channel-like protein 1
LRTOMT: Leucine-rich transmembrane and O-methyltransferase domain-containing
ILDR1: Immunoglobulin-like domain-containing receptor 1
MARVELD2: MARVEL Domain Containing
OTOF: Otoferlin
RDX: Radixin
LOXHD1: Lipoxygenase Homology PLAT Domains 1
COL11A2: Collagen Type XI Alpha 2 Chain
NCOA3: Nuclear receptor coactivator
TBX3: T-box transcription factor
POU5F1: Putative POU domain, class 5, transcription factor 1B
SALL4: Sal-like protein 4
NR0B1: Nuclear receptor subfamily 0 group B member 1
TFCP2L1: Transcription factor CP2-like protein 1
POU5F1: POU domain, class 5, transcription factor 1
NANOG: Homeobox protein NANOG
KLF4: Krueppel-like factor 4
SOX2: SRY-Box Transcription Factor 2

## References

[1] Morton CC, Nance WE (2006). Newborn hearing screening—a silent revolution. New Engl J Med.

[2] Van Eyken E, Van Camp G, Van Laer L (2007). The complexity of age-related hearing impairment: contributing environmental and genetic factors. Audiol Neurotol.

[3] Ouyang XM, Yan D, Yuan HJ, Pu D, Du LL, Han DY (2009). The genetic bases for non-syndromic hearing loss among Chinese. J Hum Genet.

[4] Tekin M, Arnos KS, Pandya A (2001). Advances in hereditary deafness. The Lancet.

[5] Shearer AE, Eppsteiner RW, Booth KT, Ephraim SS, Gurrola J, Simpson A (2014). Utilizing ethnic-specific differences in minor allele frequency to recategorize reported pathogenic deafness variants. Am J Hum Genet.

[6] Tekin M, Duman T, Boğoçlu G, Incesulu A, Cin S, Akar N (2003). Moderate hearing loss and pseudodominant inheritance due to L90P/35delG mutations in the GJB2 (connexin 26) gene. Genet Counsel.

[7] Babanejad M, Fattahi Z, Bazazzadegan N, Nishimura C, Meyer N, Nikzat N (2012). A comprehensive study to determine heterogeneity of autosomal recessive nonsyndromic hearing loss in Iran. Am J Med Genet A.

[8] Yazdanpanahi N, Chaleshtori MH, Tabatabaiefar MA, Noormohammadi Z, Farrokhi E, Najmabadi H (2012). Two novel SLC26A4 mutations in Iranian families with autosomal recessive hearing loss. Int J Pediatr Otorhinolaryngol.

[9] Fattahi Z, Shearer AE, Babanejad M, Bazazzadegan N, Almadani SN, Nikzat N (2012). Screening for MYO15A gene mutations in autosomal recessive nonsyndromic, GJB2 negative Iranian deaf population. Am J Med Genet A.

[10] Sloan-Heggen CM, Babanejad M, Beheshtian M, Simpson AC, Booth KT, Ardalani F (2015). Characterising the spectrum of autosomal recessive hereditary hearing loss in Iran. J Med Genet.

[11] Alasti F, Sanati MH, Behrouzifard AH, Sadeghi A, De Brouwer AP, Kremer H (2008). A novel TECTA mutation confirms the recognizable phenotype among autosomal recessive hearing impairment families. Int J Pediatr Otorhinolaryngol.

[12] Hashemzadeh Chaleshtori M, Simpson M, Farrokhi E, Dolati M, Hoghooghi Rad L, Amani GS (2007). Novel mutations in the pejvakin gene are associated with autosomal recessive non-syndromic hearing loss in Iranian families. Clin Genet.

[13] Davoudi-Dehaghani E, Zeinali S, Mahdieh N, Shirkavand A, Bagherian H, Tabatabaiefar MA (2013). A transversion mutation in non-coding exon 3 of the TMC1 gene in two ethnically related Iranian deaf families from different geographical regions; evidence for founder effect. Int J Pediatr Otorhinolaryngol.

[14] Taghizadeh SH, Kazeminezhad SR, Sefidgar SAA, Yazdanpanahi N, Tabatabaeifar MA, Yousefi A (2013). Investigation of LRTOMT gene (locus DFNB63) mutations in Iranian patients with autosomal recessive non-syndromic hearing loss. Int J Mol Cell.

[15] Mehrjoo Z, Babanejad M, Kahrizi K, Najmabadi H (2015). Two novel mutations in ILDR1 gene cause autosomal recessive nonsyndromic hearing loss in consanguineous Iranian families. J Genet.

[16] Grillet N, Schwander M, Hildebrand MS, Sczaniecka A, Kolatkar A, Velasco J (2009). Mutations in LOXHD1, an evolutionarily conserved stereociliary protein, disrupt hair cell function in mice and cause progressive hearing loss in humans. Am J Hum Genet.

[17] Chen W, Kahrizi K, Meyer NC, Riazalhosseini Y, Van Camp G, Najmabadi H (2005). Mutation of COL11A2 causes autosomal recessive non-syndromic hearing loss at the DFNB53 locus. J Med Genet.

[18] Ołdak M. Next Generation Sequencing in Vision and Hearing Impairment. In: Clinical Applications for Next-Generation Sequencing. Elsevier; 2016: 153–170.

[19] Moteki H, Azaiez H, Booth KT, Shearer AE, Sloan CM, Kolbe DL (2016). Comprehensive genetic testing with ethnic-specific filtering by allele frequency in a Japanese hearing-loss population. Clin Genet.

[20] Panahi Y, Fattahi A, Zarei F, Ghasemzadeh N, Mohammadpoor A, Abroon S (2018). Next-generation sequencing approaches for the study of genome and epigenome toxicity induced by sulfur mustard. Arch Toxicol.

[21] Khalili M (2015). A comparative study of ethnic identity among Azerbaijani speakers in the Islamic Republic of Iran and the Republic of Azerbaijan. Ritsumeikan J Asia Pac Stud.

[22] Kenneson A, Braun KVN, Boyle C (2002). GJB2 (connexin 26) variants and nonsyndromic sensorineural hearing loss: a HuGE review. Genet Med.

[23] Ghasemnejad T, Khaniani MS, Zarei F, Farbodnia M, Derakhsahan SM (2017). An update of common autosomal recessive non-syndromic hearing loss genes in Iranian population. Int J Pediatr Otorhinolaryngol.

[24] Saadat M, Ansari-Lari M, Farhud D (2004). Short report consanguineous marriage in Iran. Ann Hum Biol.

[25] Collin RW, Kalay E, Tariq M, Peters T, van der Zwaag B, Venselaar H (2008). Mutations of ESRRB encoding estrogen-related receptor beta cause autosomal-recessive nonsyndromic hearing impairment DFNB35. Am J Hum Genet.

[26] Festuccia N, Owens N, Esrrb PJFL (2018). Esrrb, an estrogen-related receptor involved in early development, pluripotency, and reprogramming. FEBS Lett.

[27] Wurtz J-M, Bourguet W, Renaud J-P, Vivat V, Chambon P, Moras D (1996). A canonical structure for the ligand-binding domain of nuclear receptors. Nat Struct Biol.

[28] Saïd MB, Ayedi L, Mnejja M, Hakim B, Khalfallah A, Charfeddine I (2011). A novel missense mutation in the ESRRB gene causes DFNB35 hearing loss in a Tunisian family. Eur J Med Genet.

[29] Ansar M, ud Din MA, Arshad M, Sohail M, Faiyaz-Ul-Haque M, Haque S. et al. A novel autosomal recessive non-syndromic deafness locus (DFNB35) maps to 14q24.1–14q24.3 in large consanguineous kindred from Pakistan. Eur J Hum Genet. 2003;11(1):77–80. 10.1038/sj.ejhg.5200905 PMC2917542 12529709

[30] Lee K, Khan S, Ansar M, Santos-Cortez RLP, Ahmad W, Leal SM (2011). A novel ESRRB deletion is a rare cause of autosomal recessive nonsyndromic hearing impairment among Pakistani families. Genet Res Int.

[31] Ramzan M, Bashir R, Salman M, Mujtaba G, Sobreira N, Witmer PD (2020). Spectrum of genetic variants in moderate to severe sporadic hearing loss in Pakistan. Sci Rep.

[32] Brožková DŠ, Laštůvková J, Machalová E, Lisoňová J, Trková M, Seeman P (2012). DFNB35 due to a novel mutation in the ESRRB gene in a Czech consanguineous family. Int J Pediatr Otorhinolaryngol.

[33] Wu C-C, Lin Y-H, Liu T-C, Lin K-N, Yang W-S, Hsu C-J (2015). Identifying children with poor cochlear implantation outcomes using massively parallel sequencing. Medicine.

[34] Yang T, Wei X, Chai Y, Li L, Wu H (2013). Genetic etiology study of the non-syndromic deafness in Chinese Hans by targeted next-generation sequencing. Orphanet J Rare Dis.

[35] Chouchen J, Tlili A (2020). Two new mutations, ESPN c. 2257T>C and ESRRB c. 10583C>A, cause hearing loss in UAE families. Hamdan Med J.

[36] Oza AM, DiStefano MT, Hemphill SE, Cushman BJ, Grant AR, Siegert RK (2018). Expert specification of the ACMG/AMP variant interpretation guidelines for genetic hearing loss. Hum Mutat.

