# Supplementary figures and images for: A novel missense variant in ESRRB gene causing autosomal recessive non-syndromic hearing loss: in silico analysis of a case
Source: BMC Med Genomics. 2022 Feb 1;15:18. doi: 10.1186/s12920-022-01165-4 (PMC8805370; doi:10.1186/s12920-022-01165-4)

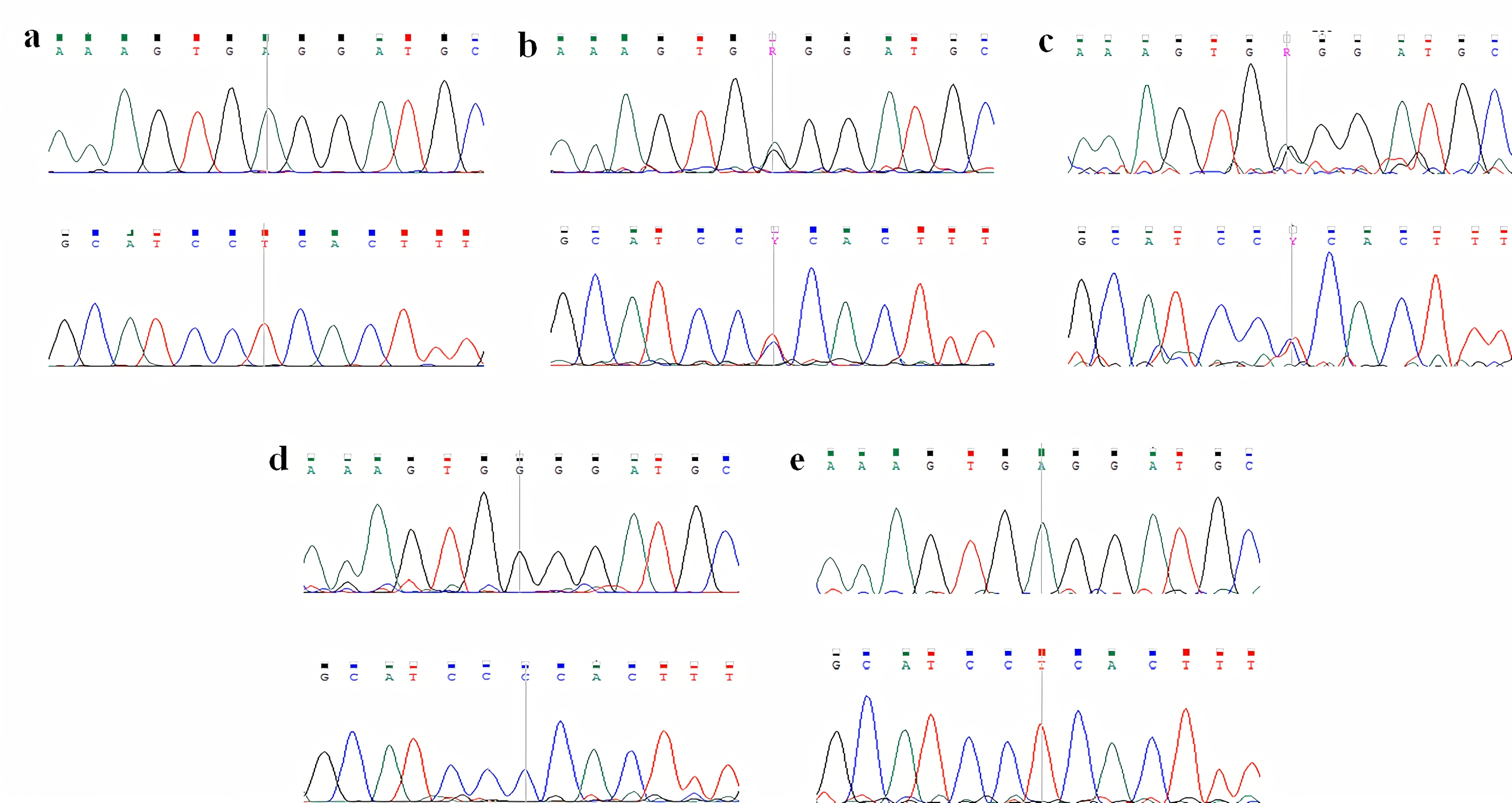

Supplement: Supplementary file 1 — Additional file 1: Forward and reverse reads obtained from sequencing of the (a) proband (V.4) (b) father (IV.6) (c) mother (IV.5) and siblings which include (d) affected sister (V.5) and (e) unaffected brother (V.2). [file 12920_2022_1165_MOESM1_ESM.png]
